# Spectroscopic technologies and data fusion: Applications for the dairy industry

**DOI:** 10.3389/fnut.2022.1074688

**Published:** 2023-01-11

**Authors:** Elena Hayes, Derek Greene, Colm O’Donnell, Norah O’Shea, Mark A. Fenelon

**Affiliations:** ^1^University College Dublin (UCD) School of Biosystems and Food Engineering, University College Dublin, Dublin, Ireland; ^2^Teagasc Food Research Centre, Moorepark, Fermoy, Ireland; ^3^University College Dublin (UCD) School of Computer Science, University College Dublin, Dublin, Ireland

**Keywords:** dairy processing, chemometrics, spectroscopy, milk, data fusion, dairy

## Abstract

Increasing consumer awareness, scale of manufacture, and demand to ensure safety, quality and sustainability have accelerated the need for rapid, reliable, and accurate analytical techniques for food products. Spectroscopy, coupled with Artificial Intelligence-enabled sensors and chemometric techniques, has led to the fusion of data sources for dairy analytical applications. This article provides an overview of the current spectroscopic technologies used in the dairy industry, with an introduction to data fusion and the associated methodologies used in spectroscopy-based data fusion. The relevance of data fusion in the dairy industry is considered, focusing on its potential to improve predictions for processing traits by chemometric techniques, such as principal component analysis (PCA), partial least squares regression (PLS), and other machine learning algorithms.

## 1. Introduction

**FIGURE 1:**
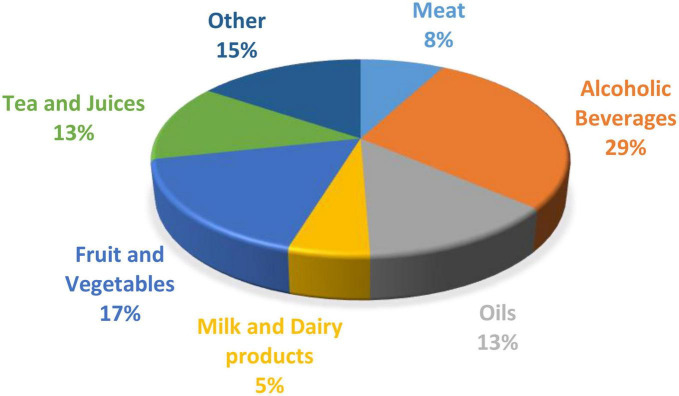
The percentage of publications on data fusion in the food industry based on each sector (6).

Sustainability and traceability are growing concerns for consumers who are continuously informed about climate change and food security systems (1). Growing consumer awareness about product quality and authenticity has led to an increased need for fast, non-invasive analytical methods (2). Many traditional analysis methods in this area are time-consuming and often require chemicals that can negatively impact the environment. Spectroscopy has become a commonly used technique due to its ease of use and application across a wide range of food nutrients, and the availability of powerful downstream chemometric tools for data interpretation. Different spectroscopy techniques have been applied to measure composition, authenticity (3), adulteration (4), physicochemical (5), and organoleptic characteristics in dairy applications. However, each method is limited in the information it can provide, often leading to poor or inaccurate calibrations. Combining multiple data sources through data fusion can provide complementary information thus increasing robustness of prediction models. While data fusion is used in the food industry, there is relatively little research reported on the application of this technique for milk and dairy products (Figure 1). This paper is structured into four sections. (Section “2. Data fusion”) introduces the topic of data fusion and the types of data fusion that are commonly used. (Section “3. Downstream method used with data fusion”) considers the downstream methods used prior to data fusion. (Section “4. Spectroscopic technologies and their usage in the dairy industry”) discusses three spectral technologies used in the dairy industry, i.e., Infrared, Raman and Fluorescence spectroscopy, and finally (see the Section “5. Applications of data fusion for the dairy industry) examines the applications of data fusion to the dairy industry. Table 1 summarizes the allocation of studies per section.

**TABLE 1:** Division of references used per section.

Section	Sub heading	References
Section 1	Data fusion	(1–14)
Section 2	Downstream methods used in data fusion	(3, 7, 15–33)
Section 3	IR	(31, 34–53)
	Raman	(54–68)
	Fluorescence	(69–87)
Section 4	Applications of data fusion for the dairy industry	(3, 6, 27, 30, 49, 53, 88–100)

Search methods used were Web of Science, Google Scholar. Key words used in search were data fusion, dairy, spectroscopy, information fusion, multi-block, milk.

## 2. Data fusion

Data fusion refers to the process of combining multiple data sources, typically to increase the accuracy and precision of downstream predictive models. It has become a popular method in the food industry in recent years due to the increased use of various spectroscopic analysis techniques. Each spectral technique has unique measurement capability that when combined, provide additional compositional information compared to when used individually. Such methods have been used extensively in remote sensing (7) and bioinformatics (8). There are many alternative data fusion strategies, varying in terms of their complexity and approach to combining data. The main challenges associated with data fusion revolve around finding an appropriate technique for integrating heterogeneous data from multiple complex systems. For instance, combining data from multiple instruments, especially in the spectroscopy area, can lead to greater noise levels during subsequent data analysis. The selection of an appropriate fusion technique is usually case-dependent and can vary greatly depending on the nature of the dataset. In many studies, different data fusion techniques are evaluated empirically, and these results are then compared to individual source results to determine the optimum technique for model development.

Many studies have shown that data fusion enhances classification and prediction performance compared to relying on individual sources (6). A review of information fusion in the food industry reported that in 81% of articles, fusion methods positively affected results, with only 2% of articles cited as having negative effects compared to non-fusion methods (9). Combining the datasets for different spectroscopic techniques, and harnessing the complementary information provided by each source suggests that it could be possible to improve calibration models in cases where one spectroscopic approach alone currently yields poor predictions.

**FIGURE 2:**
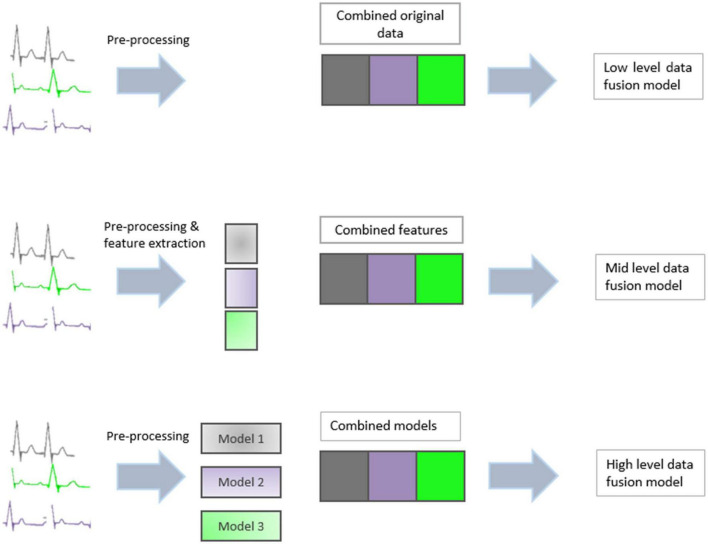
Graphical summary of three types of data fusion: Low level, mid level, and high level.

Data fusion techniques can generally be divided into three categories: low-level, mid-level, and high-level (10). A graphical summary of the three methods can be seen in Figure 2.

### 2.1. Low-level data fusion

Involves concatenating the entire dataset from each source into a new single dataset, on which a model is subsequently built. Low-level fusion is the simplest method, as it does not require the application of any feature extraction or variable reduction technique. However, it does require scaling to ensure all data blocks present with equal variance (11). A high volume of data, often containing similar or correlated information is also a limitation of low level data fusion (12).

### 2.2. Mid-level data fusion

Feature fusion reduces the dimensionality of each data source before combining the resulting information. Usually, the reduced dimensions take the form of PCA vectors or PLS latent variables. Feature-level data extraction is used mainly with mid-level data fusion. This involves taking features from different datasets and then treating them equivalently. The resulting features are concatenated into a single feature vector that is then used in classification or regression analysis.

Mid level data fusion has advantages over low level data fusion in that it can remove unwanted information through dimension reduction techniques. Variable selection techniques are also another way of selecting only relevant data from each dataset thus reducing noise and unwanted information (12). The variable reduction also reduces the computational time for analysis.

### 2.3. High-level data fusion

Decision fusion builds a prediction model for each dataset individually and subsequently combines the individual outputs to produce a single consensus prediction. High level data fusion often outperforms mid and low level data fusion as it removes unwanted data while including all relevant data. Mid level and high level data fusion gave better classification performance than those produced on individual datasets (13).

### 2.4. Multi-block methods

Multi-view or multi-block methods combine data from several datasets to provide complementary information that can be used to describe objects or images more accurately. Most multi-block approaches combine data from sources and add an X layer (where X is a matrix of variables) to the data, often known as an augmented layer, which contains the X data from the combined sources (14). We can then generate model predictions based on either the augmented model or make comparisons between each data group. Methods commonly applied in conjunction with multi-block regression include sequential and orthogonal partial least squares regression (SO-PLS), parallel and orthogonal partial least squares regression (PO-PLS), and canonical correlation analysis (15). SO-PLS is suitable when there is a pattern or order associated with the data blocks, while PO-PLS is used when equal importance is assigned to each data block (14). Multi block component analysis improved discrimination between different types of cheeses combined with feature level data fusion (16). While block scaling is often recommended for data fusion, sometimes it can have a worse effect on results than other pre-processing methods (17). It is therefore important to try various methods in order to optimize the model. This is one of the biggest disadvantages associated with data fusion analysis.

Multi block methods have similar approaches to data processing as traditional multivariate analysis however, with adaptations. Common methods used to identify common components among data sets include consensus PCA, multivariate curve resolution, hierarchical PCA, common component and specific weight analysis. These methods are limited as they do not provide unique information about each dataset (18). Methods focused on identifying common and distinct components include distinct and common simultaneous component analysis, generalized singular value decomposition, canonical correlation analysis, ComDim and variable importance in projection.

A different method that retains each data block’s original dimensionality is coupled matrix and tensor factorization. Tensors are a generalized matrices and can be seen as multi-dimensional arrays (19). This method is mostly suitable for high dimensional data, as it eliminates the need to unfold the data into a matrix and thus lose its original dimensionality. Therefore, it is a suitable method for spectral data that could often consist of more than two dimensions of data, such as time dependent spectra. The main purpose of matrix factorization is to extract features from each data set (20). One of the main limitations in coupled matrix factorization is the inclusion of both shared and unshared data in each data block.

## 3. Downstream methods used with data fusion

### 3.1. Pre-processing

Spectroscopic data is usually pre-processed to reduce the effects of noise and to enhance hidden or overlapping peaks. Pre-processing generally results in better downstream prediction models, removing scatter effects and eliminating baseline shifts. Savitzky–Golay smoothing, derivatives, detrending and multiplicative scatter correction are all common pre-processing techniques used in chemometrics. Normalization methods include auto scaling, vector normalization, standard normal variate (SNV), min-max normalization or concentration normalization (12). Normalization methods can be seen as column wise treatments and are useful for ensuring equal weight is given to each variable among the different datasets (21). In order to prevent bias between variables of different datasets, it is important to weight each data block correctly. For example, combining a spectral dataset, which has a large number of variables with data such as pH, could cause significant bias issues due to the dimension difference between data blocks. Methods such as SO-PLS and PO-PLS have been developed to cope with such dimensional differences (21). SO-PLS is not affected by scaling of each block as it is independent of the scaling, therefore is not affected by variance differences between data blocks (22).

Variable selection (also called feature selection) is another technique often used in spectroscopic data to reduce the number of variables used to represent a dataset, often improving predictive accuracy and algorithm scalability. Variable selection, used in mid level data fusion, is performed using either some automated selection criterion or manually based on the wavelengths of interest (23). In the latter case, knowledge of the sample’s chemical makeup is essential to prevent possible important information from being removed from the spectra. An understanding of the chemical nature of the sample is also important when interpreting the results. PLS and PCA are the most commonly used methods for variable selection and dimension reduction. Interval PLS (iPLS) is a modification of PLS and can be used as a variable selection method, which divides the wavelengths into a user-defined number of intervals. Based on the root mean square error of prediction (RMSEP) and lowest number of latent variables, the algorithm then selects the interval to be used in the model. This method is restrictive as it determines only one interval region, potentially removing useful information or retaining noisy regions. Adaptations of iPLS include backward iPLS and sequential iPLS. Both methods work similarly to iPLS, but allow more than one interval region to be selected. The limitations of these methods are that all parameters are user-defined, and for high volumes of data, the process can be computationally costly. Convolutional neural networks (CNN) can also be used for feature extraction (24). However, this has mainly been used with hyperspectral imaging data (25). The main advantage of CNN is the reduction of pixels from hyperspectral image data, and thus a reduction in computation.

An investigation into the use of different pre-processing techniques with data fusion was carried out by Mishra et al. (26). The authors argued that different measurement techniques can provide different levels of precision and information but will typically include unwanted variation. In spectroscopic data, this unwanted information often comes in the form of baseline shifts, light scattering, and noise from an instrument or the environment, and heterogeneity in the sample state. Several pre-processing methods are used to remove this unwanted information, and some have already been discussed in this article; however, to date, there is no gold standard for pre-processing this form of data. When carrying out pre-processing, there is a risk that some useful information will be lost or irrelevant data will not be removed. A fusion of pre-processing methods was conducted to allow all complementary information from each technique to be combined and used together (26). The same researchers further validated their claims by showing that a fusion of scatter correction techniques on near infrared spectroscopy (NIR) spectra led to improved prediction models for tablet properties (27).

### 3.2. Classification and clustering methods

Data fusion is often used for classification or discrimination purposes, where labeled data is available. However, when performing exploratory analysis, clustering methods can be employed in conjunction with data fusion by grouping data based on similar information without the requirement for any supervision (28, 29). Some of the common methods used for data fusion with machine learning are discussed (30). It highlights some of the main challenges of dealing with large amounts of data from different sources. However, clustering techniques do not work well when there is a significant level of noise in the dataset (e.g., due to inaccurate measurements). The most widely adopted clustering approach is the standard *k*-means algorithm, which attempts to group the data into *k* clusters using an iterative centroid-based approach. This approach is commonly used for hyperspectral imaging analysis (31). The user must pre-specify the number of desired clusters, which can be seen as one of the primary disadvantages of this technique. Various modifications to this approach are also often used depending on the dataset (e.g., the use of different distance measures or initialization strategies). Unsupervised clustering methods such as PCA and k-means have the potential to be used as screening tools in the dairy industry. FTIR combined with different clustering techniques was used to detect atypical milk prior to processing (32). While the clustering methods used successfully identified atypical milk, the researchers acknowledged that an unsupervised method for screening relies on a “typical” milk spectrum for comparison. It may be possible that the screening approach could detect atypical milk that is not undesirable, such as milk that has higher than normal solids content compared to undesirable atypical milk such as milk adulterated with water. In terms of supervised methods, PLS-DA provides a dimension reduction method, similar to PCA. However, while PCA only looks at the variance in the X data (variables), PLS-DA also considers the variance in Y data (reference data) and tries to correlate X with Y. It is useful when the reference data is categorical. Other popular classification methods used in conjunction with data fusion include artificial neural networks (ANNs), *k*-Nearest Neighbors (kNN), and random forest algorithms (33). ANNs represent a widely adopted family of non-linear modeling technique used to predict outcomes based on query inputs and an annotated training set. The most widely used ANN variant is backpropagation (BPNN) which has been used to recognize patterns in various food products (6). KNN is a discriminant analysis technique that is useful for classification. KNN works by selecting K- neighbors, and tries to predict the class of an unknown sample by comparing it to its nearest neighbors. The class of the predicted sample is based on the class with the most nearest neighbors (101).

### 3.3. Regression methods

Data fusion can also be used to improve regression models to predict the composition or quantify a substance. The most commonly used technique in spectroscopy is PLS. Generally, this model works quite well and is used in many applications in the food and agricultural industry (34–36). PCR is another linear method that is based on PCA. This approach uses the principal components from PCA as predictor variables and fits a linear regression model on the components. Non-linear regression is also used in chemometrics with stepwise regression, ridge regression, elastic net, and LASSO regression among the more commonly used examples (37). These methods are helpful when there is multicollinearity present in the data (38).

## 4. Spectroscopic technologies and their usage in the dairy industry

### 4.1. Infrared spectroscopy

Infrared (IR) spectroscopy is a secondary analysis method, which relies on calibration models as a quantification method. IR spectroscopy measures bond vibrations in molecules caused by a change in dipole at specific frequencies (39). Near-infrared and mid-infrared are the two most commonly used IR spectroscopic techniques in the dairy sector.

Near infrared spectroscopy (NIR) approaches provide an advantage over mid infrared spectroscopy (MIR) because they can use longer path lengths with easier-to-use optical equipment (40). Fourier Transformed (FT)-NIR has become popular for online and at-line process control in the dairy industry with many practical applications such as the determination of moisture, protein, fat and lactic acid (41, 42). FT-NIR has also been used by Grassi et al. (43) to monitor milk coagulation after the addition of rennet. The study found that an FT-NIR probe could successfully monitor coagulation in real time when combined with multivariate curve resolution and alternating least squares for data analysis. A NIR fiber optic probe was also used to measure carbohydrate and protein content in infant formula powder with root mean square error of prediction (RMSEP) of 1.89% under static conditions and 2.73% under motion conditions (0.15 m/s) (44). While these methods highlight the potential of NIR for process monitoring and control, lack of expert knowledge of chemometrics and data analytics still provides a challenge to the sector.

The MIR region is found between 4,000 and 400 cm^–1^. Characteristic absorption bands have been associated with major functional groups found in food (45). Numerous studies have found that the amide I region is associated with secondary structural characteristics of milk proteins (46–48).

MIR is widely used in the dairy industry to rapidly quantify milk composition (49). In many countries, MIR is used as an official method of milk quantification for protein, fat, lactose, and urea. MIR has been recently used to predict milk functionality traits with varying levels of success. Calamari et al. (50) found linear regression suitable for predicting titratable acidity in milk with an *R*^2^ value of 0.96 and RMSE of 0.09. Technological traits of buffalo milk were predicted using FTIR, including rennet coagulation time (RCT), pH and curd firming time (k20 min). Both RCT and k_20_ min had an *R*^2^_*cv*_ of 0.31 and 0.27, respectively, while pH was higher with an *R*^2^_*cv*_ of 0.76 [Manuelian et al. (102)]. Another study compared Bayesian regression with partial least squares regression (PLS) for technological traits such as RCT and curd yield and found that Bayesian ridge regression outperformed PLS for the prediction of RCT (*R*^2^ of 0.75 vs. 0.53) and curd yield (0.79 to 0.72) (36). However, Bayesian regression are usually more computationally intensive than PLS regression models.

A study on the prediction of milk coagulation properties using MIR reported an *R*^2^ value of 0.66 for titratable acidity and 0.59 for RCT using PLS regression (51). The findings of studies on the prediction of individual proteins (for example, α-casein, β -casein, β -lactoglobulin, α -lactalbumin, and lactoferrin milk) are contrasting. Luginbühl (52) reported standard error of cross-validation (SECV) values < 0.1 and *R*^2^ values greater than 0.99 for each model developed. This is higher than the values obtained by Bonfatti (53), who reported an *R*^2^ value of 0.8 for casein. Promising results for a prediction model for casein were developed by Calamari (54), in contrast to (55), who did not find predictions for casein accurate (*R*^2^ = 0.74) enough to be used for model development. However, the calibration set used by Calamari (54) consisted of 89 samples, while the study by McDermott et al. (55) used 730 samples. However, both studies results were lower than those reported by Sanchez et al. (56), who reported good predictions for casein fractions (*R*^2^ between 0.8 and 0.92). It was argued (57) that casein predictions in the previous models were based on the percentage of casein within the total protein content. Therefore, if the casein-to-protein ratio changes, the prediction models are inaccurate as the prediction is based on total protein and assumes that casein is 80% of total protein. Instead, chymosin was used to detect spectral changes correlated with casein through enzyme hydrolysis. Significant spectral variations were observed at different concentrations of casein (CN). Chymosin cleaves kappa CN at the 105–106 amino acid position causing casein micelles to coagulate (58) while whey proteins remain in solution. The study also concluded that casein concentration directly affected the coagulation of curd. Specific milk components have also been measured using MIR, for example, fatty acids and amino acids (55, 59). β CN phenotypes have recently been identified using FTIR (60). These researchers used a combination of FTIR, principal component analysis (PCA), and chemometrics to distinguish between different genetic variants of β CN in milk. This is relevant due to the increased consumer awareness of A2 milk; therefore, a tool to identify A1 or A2 milk is required to authenticate products.

### 4.2. Raman spectroscopy

Raman spectroscopy is another method used in vibrational spectroscopy to obtain information on the chemical composition of a substance. While IR spectroscopy is based on absorption, Raman is based on inelastic scattering (61). It is sensitive to interference such as fluorescence, which is often not a problem in IR spectroscopy. Advantages of Raman include high specificity with non-overlapping peaks. Raman signals of water are weak; therefore, this approach can provide useful information on liquid samples without the effects of water masking signals, which has been identified as an issue in IR spectroscopy (62). The main wavelengths used in dairy analysis with Raman are reported in Almeida et al. (63) and Batesttin et al. (64). Similar to other spectroscopic techniques, the main advantages include its non-destructive nature on samples and its rapid analysis with little sample preparation required. The most commonly used Raman techniques are summarized in Table 2.

**TABLE 2:** Various types of Raman spectroscopy and their advantages (65).

Techniques	Advantages
Dispersive Raman Spectroscopy	Suitable for liquid samples.
Fourier transform (FT) Raman spectroscopy	Reduced interference from fluorescence, high spectral resolution.
Surface-enhanced Raman Spectroscopy (SERS)	High sensitivity and specificity.
Spatially offset Raman spectroscopy	Reduce fluorescence, more effective illumination, allows for analysis of various types of samples.

Raman has been used to effectively measure fat in milk and milk products (66, 67). However, a low signal-to-noise ratio can limit its potential in low fat or fat-free products. A comparison of Raman and FTIR found while Raman was useful for measuring milk components, FTIR provided better quality results for macromolecules (68). Lactose has been successfully measured in milk using Raman (69). C-O-H bending at 1,087 cm^–1^ was used to quantify lactose with an *R*^2^ value of 0.99 based on a linear regression model. Different laser settings can also cause interference requiring pre-processing. Most of the work done in dairy products has been on milk fats, which yielded the most accurate results. Milk fat content was determined using Raman combined with PLS with low root mean square error (RMSE) (0.16) and *R*^2^ validation of 0.97 (67). A least squares fitting approach to characterize the nutritional composition of milk gave excellent correlations for fat and lactose (*r* = 0.93 and 0.91) (70). Numerous studies have used Raman to determine conjugated linoleic acid (CLA) content in milk. Three specific bands in Raman spectra were found to be related to CLA’s *cis Trans* and conjugated bonds (71). PLS and multiple linear regression (MLR) using these bands successfully predicted CLA in milk with MLR slightly out-performing PLS (*R* = 0.975 vs. 0.951). Raman has also been successfully used to detect adulterants in milk, for example, melamine in infant formula. Almeida et al. (63) explored the use of FT-Raman for milk powder screening. PCA analysis was used to separate whole milk powder, skim milk powder and adulterated powders. Partial least squares discriminant analysis (PLS-DA) models successfully classified 100% of powders adulterated with varying amounts of whey. It has been used to quantify the composition of milk powders (72, 73), i.e., PLS regression for prediction of fat and protein. The effect of temperature was investigated (72) and found that low and high temperatures tended to over- and under-predict milk fat, respectively. Some research has been conducted to examine the use of Raman to monitor milk processing. Conformational changes in whey proteins and fouling of heat exchangers using micro Raman spectroscopy were investigated (74). The amide I region at approx. 1,670 cm^–1^ was found to show differences between dry powder and aggregated powder, which can be interpreted as increases in β-turns upon heating and an intensity decrease at 940 cm^–1^ was associated with loss of alpha helix structures. However, in Raman the signal for protein is much weaker than in IR spectroscopy and therefore is not used as frequently for measuring protein.

### 4.3. Fluorescence spectroscopy

Fluorescence spectroscopy can be used to analyse the physico - chemical properties of various dairy products. It has gained popularity mainly due to improved instrumentation and advances in data analytics (multivariate and chemometric analysis). Proteins and lipids often contain specific fluorophore regions; hence, fluorescence spectroscopy can pick up small changes in their structure due to its high sensitivity. However, like other spectroscopic methods, it requires instrument standardization and validation for use at a large industrial scale. Traditional fluorescence measures fluorescent emission from fluorophores in clear solutions (75). Scattering and fluorescent quenching affect measurements of opaque or solid samples. Fluorescence quenching, a process that reduces the fluorescence intensity of a sample, could be used to characterize interactions between flavonoids and proteins in dairy ingredients (76). The study showed that fluorescence quenching was due to ligand binding between pelargonidin and proteins. Due to the nature of milk, it can be difficult to measure using a photometric method. Therefore, a different approach has been used: front-face fluorescence spectroscopy (FFFS). This technique only measures the fluorescence emitted from the sample’s surface and removes problems associated with scatter and quenching. The advantages of FFFS are that it can be used on turbid liquids and powders, and no sample preparation is required. Andersen and Mortensen (75) provide an in-depth review of the use of fluorescence spectroscopy in the analysis of dairy products. A further review on the application of fluorescence spectroscopy in dairy processing has been conducted by Shaikh and O’Donnell (77). The fluorescence in milk products mainly lies in riboflavin, vitamin A, aromatic amino acids (tryptophan, phenylalanine, and tyrosine), NADH, lipid oxidation products, and other chemical compounds that induce fluorescence emission. Most studies have focused on the fluorescence of tryptophan when measuring the protein structure in milk products. The maxima of tryptophan emission peaks were identified at 343 nm, and a direct relationship between heat treatment and fluorescence properties was illustrated by the change in emission spectra when normal ultra-high temperature (UHT) milk was compared to over-heated UHT milk (78). The denaturation of protein during heat treatment alters the tryptophan region of proteins. Fluorescence increases with increasing heat treatment in milk, mainly due to the unfolding of the protein structure, resulting in exposure of more tryptophan residues (75). Heat treatments also cause the production of Maillard reactions, which are measured at 440 nm in the emission spectrum. The study by Kulmyrzaev and Dufour (78) identified that FFFS can monitor the production of Maillard reactions, mainly lactulose and furosine, and an increase in intensity at 430 nm indicates that other fluorescent compounds are being produced in UHT milk; this was not seen in pasteurized milk. Using principal component regression, an *R*^2^ value of 0.95 was obtained for comparison of predicted versus reference furosine while an *R*^2^ = 0.987 was found for lactulose. Lactulose and furosine are not fluorophores and therefore, the correlation is by an indirect measurement between the tryptophan spectra and the concentration of lactulose and furosine. PCA can be used to monitor changes between raw milk, heated, homogenized and homogenized and heated samples (79). The first two principal components captured 96% of total variance for the tryptophan emission spectrum data and over 99% of the total variance for Vitamin A. A more recent study (80) discriminated milk based on thermal treatment using PCA. Strong correlations were found between spectra, alkaline phosphate, and β lactoglobulin using principal component regression (PCR).

Fluorescence spectroscopy has been used to detect changes in the structure of casein micelles during coagulation (81) and measure the binding properties of β-LG during folding (82). This technique provides another tool for determining, at a molecular level, the structural changes that occur during milk coagulation and heating. Fluorescence spectroscopy was used to investigate the effects of heating milk on curcumin binding to CN (83). Milk was heated to 80°C, and the fluorescence intensity of curcumin increased. Front-face fluorescence has become popular as a rapid, non-invasive method of analysis for fluorescent molecules and their interactions in biological samples.

Casein is the primary component that coagulates in milk during cheese making; therefore, a rapid quantification method can facilitate the identification of optimal coagulum cut time during manufacture. Tryptophan and riboflavin, both intrinsic fluorophores, could be used to monitor rennet-induced coagulation of milk by measuring the change in fluorescence intensity during coagulation (84). Acidification of casein can also be measured using FFFS as, when casein is in an acidic environment, it undergoes structural changes that increase the fluorescence intensity of tryptophan. This is associated with structural and conformational changes in the casein micelle as colloidal casein phosphate (CCP) dissociates from the micelle at low pH, exposing more tryptophan residues (85). In the study, casein was precipitated using acetic acid prior to fluorescence analysis. PLS and elastic net regression models both performed well in predicting casein% with *R*^2^ value of 0.91 for both PLS and elastic net and cross validated RMSE of 0.12% (85). Heat-induced coagulation was also measured using fluorescence (86). The study was based on using tryptophan as marker, where quenching was observed upon coagulation of milk. The change in tryptophan emission spectra has been related to structural changes in proteins, such as protein denaturation (86).

Front-face fluorescence spectroscopy has also been used to monitor thermal processing in milk; however, no sample preparation was required compared to previous studies (87). The authors found a strong correlation (*R*^2^ = 0.95) between FFFS and the reference method, indicating that FFFS can be used with no sample preparation for measuring thermal processes in milk. Riboflavin is also used as an indicator for lipid-induced oxidation. Miquel Becker et al. (88) used PLS with a prediction error of 0.0092 mg riboflavin/100 g yogurt to detect riboflavin in yogurt. The study demonstrated that riboflavin could be used as an early indication of degradation during storage.

Given current awareness regarding product origin and traceability in the dairy industry, a rapid technique based on fluorescence and riboflavin could be useful in confirming product authenticity. Fluorescence has also been used to authenticate milk from grass-fed cows (89). The levels of riboflavin and chlorophyll metabolites were measured in the milk and shown to be significantly higher in grass-fed cows than in grain or silage-fed cows, most likely due to the higher level of chlorophyll in fresh grass. Tryptophan and riboflavin have been used as intrinsic indices for online measurement in milk processing. The geographic origin of milk has been determined through the use of discriminant analysis using fluorescence spectroscopy (90). Although the dataset was small, the calibration model classified 100% of the samples correctly, and the validation model had a classification accuracy of 69%. In particular, the lowland samples were well separated from the upland and midland samples. The sample set is however, too small to be able to confirm how effective this method is.

The combination of data from fluorescence and MIR, which provides detailed information about the chemical composition of milk, could offer a more effective approach when developing prediction models for processing traits, such as rennet coagulation time and heat stability. This is discussed in more detail below.

## 5. Applications of data fusion for the dairy industry

Quality and authentication are two important concepts in the dairy industry. Products are valued based on the quality of ingredients and often from the origin of where they are produced. Adulteration, therefore, is an issue in the dairy industry. Food adulteration is the addition of cheaper materials into a food product to increase the amount of the product or to increase specific components. One of the most well known cases of adulteration in the dairy sector occurred in 2008 in China where melamine was used to increase the nitrogen content of milk. The milk was used in the manufacture of infant formula and resulted in over 50,000 babies becoming seriously ill (91). This, and other incidents, highlight the need for rapid detection methods.

Spectroscopy has been a widely used method for milk compositional analysis (59, 92–94) and for detecting product adulteration over the last few years (95). There are many benefits of spectroscopy compared to traditional analytical techniques, as it is fast, non-destructive, requires no harsh chemicals, and is cost-effective. However, each spectroscopy technique has its limitations, as discussed previously. It is an analytical measurement that relies on a calibration model for compositional or classification analysis. Combining spectra from different wavelength regions has proven in some cases to be more accurate than individual spectra from one region (6, 96). In recent years data fusion has been adopted as a novel method to increase prediction accuracy for classification and regression analysis in the food industry (11, 97). The distribution (%) of publications on data fusion for each sector of the food industry is shown in Figure 1. The area with the highest number of publications incorperating data fusion techniques is alcoholic beverages, such as beer and wine.

Providence is an important consideration for the dairy sector, and the cows’ diet influences the levels of constituents in milk responsible for its authentication. Due to increasing demands for improved sustainability, forage-based diets are considered environmentally friendly and better for animal welfare.

Thus, the use of animal diet to discriminate between milk from different regions can provide a valuable tool for the industry and consumer. O’Callaghan et al. (98) discussed the effects of pasture-based diets on milk metabolomics, which can be used to identify the diet type of a cow. A study by Riuzzi (99) used mid-level data fusion to authenticate milk samples from different forage-based diets. With the growing demand for traceability, this analysis could provide an accurate method for milk authentication. Data fusion was used to improve the discrimination ability of PCA to identify milk that has been fortified with milk powder (100). Electronic tongue and nose were used to distinguish between UHT and pasteurized milk combined with the use of PCA (103).

A comparative study (104) applied PLS and support vector machine (SVM) on full spectra and wavelength-selected spectra for Vis-NIR and Raman data. Compared individual models with data fusion models combining both Vis-NIR and Raman for the discrimination of storage time and temperature on infant formula. Low, mid, and high level data fusion models were compared in each case. In the case of storage temperature discrimination, the full spectral dataset for Vis-NIR using SVM was the most effective model while mid-level data fusion using SVM produced the best model for storage time. Zhao (105) compared laser induced breakdown spectroscopy (LIBS), FTIR and Raman prediction models for quantification of calcium in infant formula. Low and mid level data fusion methods were also compared. In this case the prediction model developed from LIBS was the most accurate with an *R*^2^_*cv*_ = 0.99, while the mid level data fusion model achieved *R*^2^_*cv*_ = 0.97.

Milk processing is an integral part of product development. Heating, drying, and processing milk affects its structure, affecting the quality or development of milk-derived products. Many studies have tried to develop calibrations for NIR and MIR instruments to predict processability traits such as heat stability and rennet coagulation. However, the calibration models using PLS have been unsuccessful (35, 55, 106). The possibility of data fusion, combined with other chemometric and machine learning techniques, could allow these processability traits to be accurately predicted. The increased use of spectral sensors in process unit operations increases the possibility for using data fusion methods in dairy manufacturing facilities.

A novel approach to monitor milk processing used a combination of raw and first derivative spectra with autoencoder neural networks to detect changes in milk during processing (107). An auto-encoder was trained using 1.5% UHT milk. The combined data improved anomaly detection of fat, temperature and production compared to either data set; however, the raw spectra alone proved more accurate for detecting water or cleaning solution in the milk. This method was used during processing, and such techniques would allow for early detection of abnormal changes and prevent problems further down the processing line. This provides an advantage to the processor by reducing the need for laborious analytical methods. However, continuous maintenance of data fusion calibrations is required to avoid inaccurate measurements.

## 6. Conclusion/Final remarks

Data fusion has been demonstrated in various settings as providing more accurate predictions compared to using one data source. In particular, accurate models are needed for milk analysis and processability, authenticity, quality and adulteration due to the increased awareness around food traceability. Numerous analytical methods are used to create data fusion models, and the optimal technique is often sample dependent, as there is no “one-size-fits-all” approach. Pre-processing is usually required prior to fusion, to remove noise, reduce variables and scale data blocks. However, it is important not to excessively pre-process the data to a point where valuable information is lost. For difficult-to-measure traits such as RCT, heat stability and other milk processing traits, data fusion could provide further benefits by combining complementary information from different spectral technologies, leading to increased prediction accuracy. While data fusion can provide rapid and accurate measurement, the initial calibration and model development is time consuming and requires expert knowledge of sample chemistry and machine learning.

## Data availability statement

The original contributions presented in this study are included in the article/supplementary material, further inquiries can be directed to the corresponding author.

## Author contributions

MF contributed to revisions and the dairy processing section. CO’D contributed to revisions. DG contributed to the data fusion section and revisions. NO’S contributed to the spectroscopy section and revisions. All authors contributed to the article and approved the submitted version.
